# Mass spectrometry screening reveals widespread diversity in trichome specialized metabolites of tomato chromosomal substitution lines

**DOI:** 10.1111/j.1365-313X.2010.04154.x

**Published:** 2010-03-08

**Authors:** Anthony Schilmiller, Feng Shi, Jeongwoon Kim, Amanda L Charbonneau, Daniel Holmes, A Daniel Jones, Robert L Last

**Affiliations:** 1 Department of Biochemistry and Molecular Biology, Michigan State UniversityEast Lansing, MI 48824, USA; 2 Department of Chemistry, Michigan State UniversityEast Lansing, MI 48824, USA; 3 Department of Plant Biology, Michigan State UniversityEast Lansing, MI 48824, USA; 4 Department of Energy Plant Research Laboratory, Michigan State UniversityEast Lansing, MI, USA

**Keywords:** specialized metabolism, secondary metabolites, introgression lines, terpenes, solanum, time of flight mass spectrometry

## Abstract

Glandular secreting trichomes of cultivated tomato (*Solanum lycopersicum*) and close relatives produce a variety of structurally diverse volatile and non-volatile specialized (‘secondary’) metabolites, including terpenes, flavonoids and acyl sugars. A genetic screen is described here to profile leaf trichome and surface metabolite extracts of nearly isogenic chromosomal substitution lines covering the tomato genome. These lines contain specific regions of the *Solanum pennellii* LA0716 genome in an otherwise ‘wild-type’ M82 tomato genetic background. Regions that have an impact on the total amount of extractable mono- and sesquiterpenes (IL2-2) or only sesquiterpenes (IL10-3) or specifically influence accumulation of the monoterpene α-thujene (IL1-3 and IL1-4) were identified using GC-MS. A rapid LC-TOF-MS method was developed and used to identify changes in non-volatile metabolites through non-targeted analysis. Metabolite profiles generated using this approach led to the discovery of introgression lines producing different acyl chain substitutions on acyl sugar metabolites (IL1-3/1-4 and IL8-1/8-1-1), as well as two regions that influence the quantity of acyl sugars (IL5-3 and IL11-3). Chromosomal region 1-1/1-1-3 was found to influence the types of glycoalkaloids that are detected in leaf surface extracts. These results show that direct chemical screening is a powerful way to characterize genetic diversity in trichome specialized metabolism.

## Introduction

Secreting glandular trichomes (SGTs) are epidermal protuberances that are found on a wide variety of plant species (Wagner, 1991; Schilmiller *et al.*, 2008). These structures manufacture large amounts of specialized (also known as secondary) metabolites, and either store these molecules in the extracellular space or deposit them on the epidermal surface, sometimes in amounts sufficient to cause stickiness to the touch. Trichome compounds such as those in basil (Iijima *et al.*, 2004), mint (Croteau *et al.*, 2005) and hops (Nagel *et al.*, 2008) are responsible for their smell and taste. Other plants manufacture compounds of medicinal importance, such as the anti-nausea agent tetrahydrocannabinol from *Cannabis sativa* (Sirikantaramas *et al.*, 2005) and the anti-malarial agent artemisinin from wormwood (Duke *et al.*, 1994). The combination of active biosynthesis, the species specificity of metabolite accumulation and the ease of purification of trichomes makes them an excellent system for elucidation of specialized metabolite pathways and identification of genes encoding the pathway enzymes (Wagner, 1991; Schilmiller *et al.*, 2008).

Forward genetics (screening for an altered phenotype in a collection of genetically diverse individuals) provides a complementary approach for the discovery of enzymes, transporters or regulators of poorly defined biosynthetic pathways or primary sequences not recognizable by homology. One of the advantages to this approach is that, by directly screening genetic variants for changes in metabolite levels, it is possible to identify genes encoding completely novel products (Benning, 2004). Although GC- and LC-based methods have been used successfully to identify plant biochemical mutants (Browse *et al.*, 1985; Sattler *et al.*, 2003; Valentin *et al.*, 2006; Kliebenstein *et al.*, 2007), the combination of GC or LC with MS is an especially powerful approach to screen for phenotypic variants (Jander *et al.*, 2004; Schauer *et al.*, 2006; Lu *et al.*, 2008) because of the analytical sensitivity, specificity and amount of information provided.

The combination of mutagenesis and genetic mapping is a tried and true approach for identification of genes that cause changes in metabolite accumulation in tomato and other model plant species (for representative examples regarding tomato fruit and flower color, see Galpaz *et al.*, 2006; Isaacson *et al.*, 2002 and Ronen *et al.*, 2000). However, screening naturally occurring genetic variants is an increasingly popular approach due to recent advances in genomics technologies that simplify the identification of causative genes (Rounsley and Last, 2010). An advantage of using natural variation for gene discovery is that it is far more likely to yield examples of gain-of-function alleles than use of mutagenesis, which favors loss-of-function mutations (van der Hoeven *et al.*, 2000; Sallaud *et al.*, 2009; Schilmiller *et al.*, 2009).

As part of a project that aims to characterize the metabolic diversity found in SGTs of cultivated tomato and related wild species, we are using forward genetics strategies to discover genes that affect specialized metabolites. We describe here the results of screening M82 ×*Solanum pennellii* LA0716 chromosomal substitution introgression lines (ILs) using GC-MS to measure terpenes, in parallel with a rapid LC-TOF-MS method to analyze non-volatile specialized metabolites. We describe the influence of various LA0716 chromosomal segments on the accumulation of total trichome terpenes or acyl sugars, alteration of sesquiterpenes without an affect on monoterpenes, accumulation of specific molecules (the monoterpene α-thujene and acyl sucroses lacking an acetyl group) and shifts in the length of acyl chains in acyl sucrose. Together, these results demonstrate the value of using the nearly isogenic chromosome substitution lines and direct chemical characterization to explore chemical diversity in SGT metabolism.

## Results

The objective of this study was to discover regions of the *S. pennellii* LA0716 genome that modify accumulation of specialized metabolites in SGTs as a first step toward discovering gene products involved in these metabolic pathways. This study takes advantage of a series of chromosomal substitution lines that systematically replace parts of genome of the *Solanum lycopersicum* variety M82 with homologous regions from the wild species *S. pennellii* LA0716 (Eshed and Zamir, 1994, 1995). These lines were constructed in such a way that they each contain one genetically characterized region of an LA0716 chromosome in an otherwise M82 tomato genetic background. The screened lines span the entire genome with the exception of a relatively small region at the top of chromosome 5, which is unique to IL5-1 (bin 5A).

Screening of the majority of the 65 ILs was performed in 2–4-fold replication, together with 30 parental *S. lycopersicum* M82 control plants, using the leaf dip method. Leaflets of 3-week-old growth chamber-grown plants were harvested and immediately extracted by brief and gentle agitation in one of two solvents selected for extraction of a broad range of metabolites from the leaf surface, including the contents of SGT cells. While we cannot rule out the possibility that some of the chemicals found using the leaf dip method came from pavement epidermal cells or inside the leaves (particularly the glycoalkaloids), the patterns of metabolites detected are quite similar to those for trichomes scraped from the leaf surface (Schilmiller *et al.*, 2009). The chemical compositions of the ILs were compared with the isogenic M82 parent, and any changes in recognizable classes of chemicals were verified by re-growing and chemically analyzing the ILs and the M82 parent.

### GC-MS screening of ILs

GC-MS offers an easy way to detect and quantify non-polar volatile metabolites from SGTs by extraction of whole-leaf tissue in *tert-*butyl methyl ether (MTBE) (Gang *et al.*, 2001). The predominant volatile compounds detected in M82 tomato leaf dips are C10 monoterpenes and C15 sesquiterpenes produced in the trichomes (Schilmiller *et al.*, 2009), and the same quantitative and qualitative pattern was observed for the majority of ILs screened. Previous work with *Solanum habrochaites* ILs demonstrated that sesquiterpene synthase(s) on chromosome 6 control production of sesquiterpenes in *S. lycopersicum* trichomes (van der Hoeven *et al.*, 2000). Consistent with these published studies, the overlapping *S. pennellii* ILs 6-2 and 6-2-2 do not accumulate detectable sesquiterpenes in leaf trichomes, this also is the sesquiterpene phenotype of *S. pennellii* LA0716 (Figure S1).

**Figure 1:**
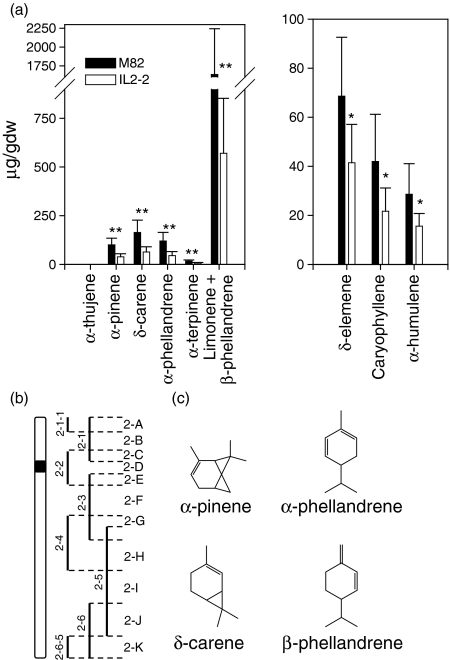
Terpene levels are reduced in IL2-2. (a) GC-MS analysis of leaf dips shows an overall decrease in terpene levels in IL2-2. Error bars represent standard deviation (*n*=10). The decrease in each compound is statistically significant at **P<0.015 or *P<0.001 (Mann–Whitney rank sum test). Similar data were also obtained in two other independent experiments with sample number ranging from 2 to 5. (b) Schematic representation of chromosome 2 introgressions showing the locus controlling low terpene levels located on bin 2-D. (c) Structures of some of the affected terpenes.

**Figure 2:**
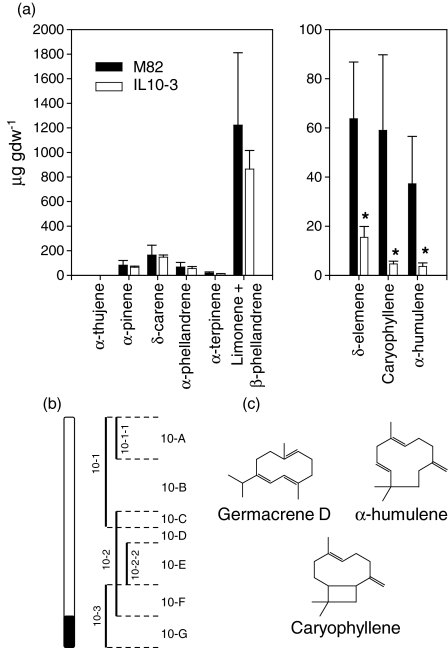
Sesquiterpenes are reduced in IL10-3. (a) GC-MS analysis of leaf dips shows a decrease in sesquiterpenes only. Error bars represent standard deviation (*n*=5). Asterisks indicate a significant difference at *P*≤0.008 (Mann–Whitney rank sum test). (b) Schematic representation of introgressions for chromosome 10. Low sesquiterpene levels are controlled by a locus on bin 10-G. (c) Structures of sesquiterpenes reduced in IL10-3. δ-elemene is a thermal degradation product of germacrene D that is produced in the hot inlet of the GC-MS.

**Figure 3:**
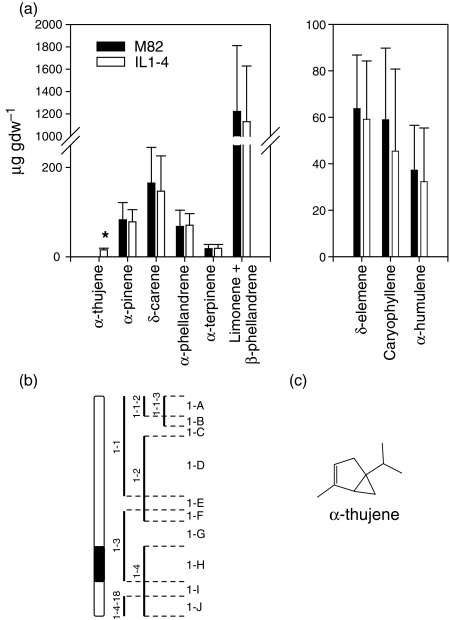
IL1-4 (shown) and IL1-3 accumulate the monoterpene α-thujene. (a) In addition to the monoterpenes normally found in *Solanum lycopersicum*, ILs 1-3 and 1-4 also accumulate detectable levels of α-thujene (indicated by an asterisk). No statistically significant differences in other terpenes were observed (Mann–Whitney rank sum test). Error bars represent standard deviation (*n*=5). (b) Schematic representation of chromosome 1 introgressions showing the locus controlling low terpene levels located on bin 1-H. (c) Structure of α-thujene.

Two other ILs were found to accumulate reduced levels of terpenes compared to M82. For IL2-2, the levels of all detected terpenes were reduced, with monoterpenes accumulating to approximately 40% of M82 levels and sesquiterpenes accumulating to approximately 65% of M82 levels (Figures 1a,c and 2c). None of the other ILs on chromosome 2 displayed this phenotype, suggesting that the locus controlling the reduction in terpene levels is in the region unique to IL2-2, defined as bin 2-D (Figure 1b). In contrast, IL10-3 showed a 75–90% reduction of sesquiterpenes alone (Figure 2a,c). Because this phenotype is specific to IL10-3, the locus controlling reduction in sesquiterpenes must be located on bin 10-G (Figure 2b). This method also allowed identification of a low-abundance monoterpene that is not found in M82 leaf dips: ILs 1-3 and 1-4 were both found to accumulate α-thujene (Figure 3a,c), a monoterpene that is present in *S. pennellii* LA0716 trichomes, but is not normally detected in M82. Because two overlapping introgressions display this phenotype, the location of the locus controlling α-thujene accumulation can be narrowed to bin 1-H, the region in common between ILs 1-3 and 1-4 (Figure 3b).

### Development of a LC-TOF-MS method for rapid profiling of trichome metabolites

Analysis of large numbers of complex biological samples for non-volatile specialized metabolites offers challenges that are either not encountered or less serious with GC-MS analysis of volatile compounds. The diversity of non-volatile specialized metabolite chemical properties and wide range of concentrations present a special set of problems for genetic screening, in which speed and reproducibility from sample to sample are desirable but conflicting properties. Direct-infusion MS (Smedsgaard, 1997; Allen *et al.*, 2003; Castrillo *et al.*, 2003; Goodacre *et al.*, 2003; Dettmer *et al.*, 2007) without chromatographic separation offers short analysis times. Unfortunately, this fast method suffers from suppression of ionization (Dettmer *et al.*, 2007), limited ability to distinguish chemical isomers, and complications that arise from in-source formation of adduct, oligomer and fragment ions due to the complexity of the mixture entering the MS. LC separation prior to MS analysis reduces the impact of these technical problems, but often involves analysis times of 30 min or more per sample, reducing its utility for screening large numbers of samples. Recent improvements in LC column technologies have provided enhanced chromatographic resolution with short run times through use of smaller particle sizes and particles with solid cores (Cunliffe *et al.*, 2007; Hsieh *et al.*, 2007; Kirkland *et al.*, 2007; Salisbury, 2008).

**Figure 4:**
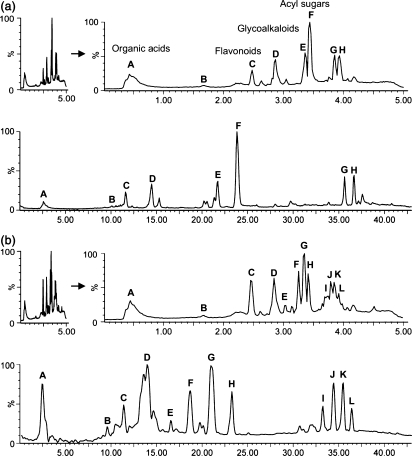
Development of a rapid LC-TOF-MS method. Total-ion chromatograms obtained from leaf dip extraction of M82 (a) and IL8-1-1 (b) for a 5 min gradient using an Ascentis Express C18 fused-core column (2.1 × 50 mm; 2.7 μm) (top) compared to a 43 min gradient using a Thermo BetaBasic C18 column (1 × 150 mm, 3 μm) (bottom) in ESI negative mode. A, malic acid and quinic acid; B, chlorogenic acid; C, rutin; D, tomatine; E–L, acyl sugars.

In the current study, we compared the metabolite profiling performance of a 5 min LC-TOF-MS method (Gu *et al.*, 2010) using an ultra-performance fused-core LC column with that of LC-MS performed using a more conventional column and a 43 min gradient. Profiles of specialized metabolites were generated for M82 and an IL using extracts from tomato leaflets dipped in solvent for selective extraction of trichome and other leaf surface metabolites (Figure 4a,b). The major observed metabolites, including malic acid, quinic acid, chlorogenic acid, the glycosylated flavonoid rutin, the glycosylated alkaloid tomatine and multiple acyl sugars, were resolved using both separation methods, with minimal sacrifice of metabolite resolution in the faster method.

A major benefit of coupling LC to TOF-MS is the fast acquisition of accurate mass measurements for metabolites in complex mixtures. This is achieved by rapid generation of mass spectra of metabolites eluting from the column over a range of mass-to-charge (*m/z*) ratios, followed by automated extraction of ion abundances that are catalogued based on retention time and *m/z* value (Wilson *et al.*, 2005). As shown in Figure S2, 15 or more acyl sugar molecules were revealed by analyzing chromatograms for 11 *m/z* ratios associated with various homologs of this class of metabolite. In some cases (e.g. *m/z* 737 and 695), multiple metabolites of the same molecular masses (structural isomers) were resolved by the LC separation, even using the 5 min LC method (Figure S2, right panel). The full dataset is provided in Table S1, and details of the data analysis used to identify ILs with alterations in specialized metabolites are described in Appendix S1.

### ILs 1-3 and 1-4 lack an acetyl group on abundant acyl sucrose metabolites

**Figure 5:**
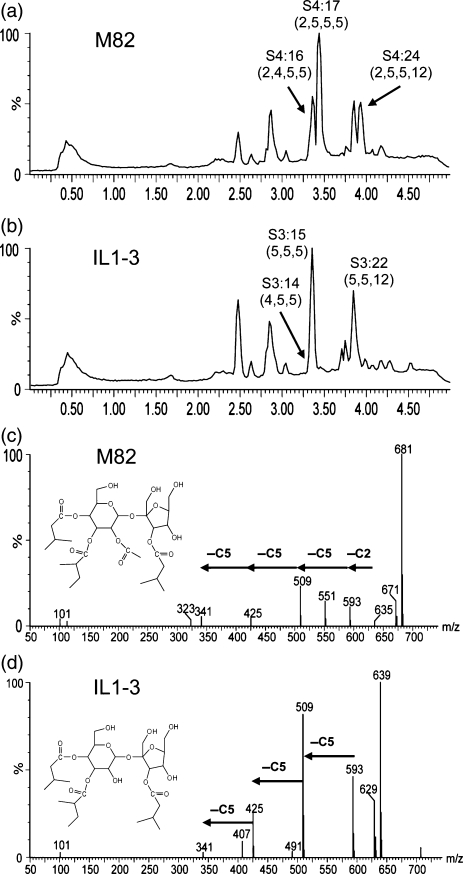
ILs 1-3 (shown) and 1-4 lack an acetyl group on major acyl sugar molecules. (a, b) Total-ion LC-MS chromatograms obtained from leaf dip of M82 (a) and IL1-3 (b). The peaks are labeled to show the disappearance of an acetyl (C2) group from IL1-3 acyl sugars. (c, d) Mass spectra obtained under elevated CID voltage (aperture 1 voltage: 55 V) are shown for peaks at retention times of 3.44 min for M82 (c) and 3.56 min for IL1-3 (d). The structure shown in (c) is based on both LC-MS and NMR data (see Appendix S1). The structure in (d) is based on LC-MS data.

Comparisons of LC-MS total-ion chromatograms for M82 with those from the overlapping ILs 1-3 and 1-4 revealed changes in the major peaks from the chromosomal substitution lines (Figure 5a,b). For example, the mass spectrum of the most prominent metabolite peak in M82, generated using gentle ionization conditions, is dominated by an ion of *m/z* 681. Mass spectra generated using a collision energy that was 15 V higher showed a peak that had a mass that was lower by 46 Da (*m/z* 635). This mass difference corresponds to formic acid, suggesting that the original ion was a complex with formate present in the HPLC mobile phase, and this is consistent with a molecular mass of 636 Da for the metabolite. However, higher collision energies generated fragment ions at masses corresponding to neutral losses of one C2 and three C5 fatty acids, and an additional fragment consistent with the C5 fatty acid anion. These findings led to the conclusion that this metabolite has one acetate ester and three C5 fatty acid ester groups (Figure 5c). The TOF-MS yielded mass measurements accurate to within 0.003 Da that supported these assignments, and were consistent with an elemental formula of C_29_H_48_O_15_ for the metabolite. The combined information from accurate measurements of molecular and fragment ion masses suggested a disaccharide substituted with the ester groups mentioned above, but MS data alone were insufficient for definitive and complete determination of the chemical structure.

NMR analysis of the purified metabolite revealed the structure shown in Figures 5(c) and S3, confirming the MS results (see Appendix S1 and Figure S3 for detailed information about NMR analysis). We refer to this acyl sugar as S4:17; in this nomenclature, ‘S’ refers to sucrose, ‘4’ indicates the total number of acyl chains, and ‘17’ is the sum of the number of carbon molecules in the acyl chains.

Two additional major tetra-acyl sucroses were also detected in M82 leaf dips, corresponding to substitution of either C4 or C12 fatty acids for one of the C5 chains (Figure 5a). We analyzed positive-ion fragmentation products resulting from cleavage of the glycosidic bond. This allowed determination of which acyl groups are substituted on the six- and five-membered rings. In both cases, the variable-length chains were found on the glucopyranose ring. A single five-carbon acyl chain was found on the five-member ring for all acyl sugars reported here.

In contrast with M82, S4:17 is barely detectable in IL1-3, for which the mass spectrum of the most prominent peak is dominated by an ion at *m/z* 639. The mass spectrum generated at elevated collision energy again suggests the presence of three C5 fatty acid esters, but no evidence for the acetate (Figure 5d). By analogy to S4:17, and based on accurate mass measurements, we infer that this metabolite is a tri-acyl sucrose with three C5 fatty acid esters, and is abbreviated as S3:15. Deacetylated variants of other tetra-acyl sucrose metabolites are also found in IL1-3 (Figure 5b).

### IL8-1-1 causes a shift in acyl chain lengths without altering the number of substitutions

**Figure 6:**
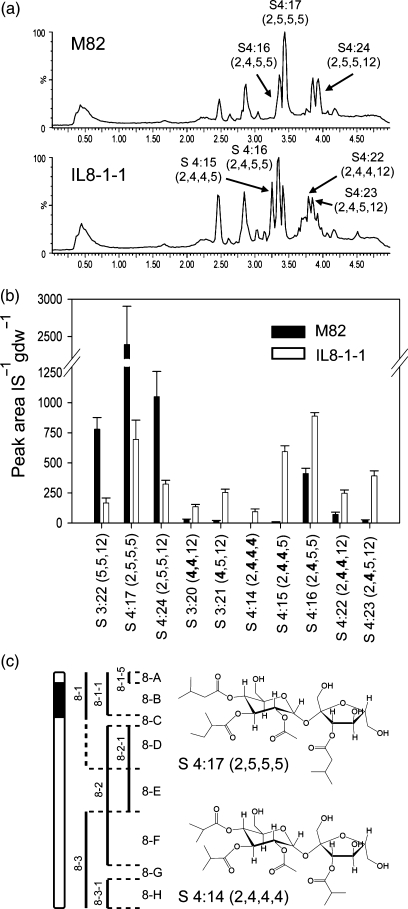
Acyl sugars in IL8-1-1 have a higher proportion of isobutyl (C4) acyl chains. (a) Total-ion chromatograms from LC-TOF-MS analysis of acyl sugars in leaf dips of M82 and IL8-1-1. Labeling nomenclature for acyl sugars: S3:22 (5,5,12) is an acyl sucrose with three acyl chains having a total of 22 carbons, and the numbers in parentheses indicate the lengths of the individual acyl chains. (b) Amounts of acyl sugars showing differences in abundance between M82 and IL8-1-1 shown as integrated peak areas normalized to the internal standard and the dry weight of the extracted leaflet. Error bars indicate standard deviation (*n*=4). (c) Schematic representation of chromosome 8 introgressions showing the locus controlling the acyl sugar phenotype located on bin 8-B. The structure of S4:17 (2,5,5,5) is based on LC-MS and NMR data. The structure of S4:14 (2,4,4,4), an acyl sugar detected only in IL8-1-1 and not in M82, is inferred based on negative and positive mode LC-MS.

The LC-MS total-ion chromatograms for IL8-1 and IL8-1-1 also showed changes in major acyl sugar peaks when compared to M82 (Figure 6a). In these lines, a major metabolite with a molecular mass 14 Da lower than that of S4:17 emerged as the dominant peak in the chromatograms. Fragmentation and accurate mass measurements indicated that this peak is a S4:16 acyl sucrose with a C4 fatty acid substituted for one of the C5 moieties (Figure 6a,b). This is a quantitative change and M82 accumulates S4:16 at approximately 50% of the level seen in IL8-1 and 8-1-1. Further analysis of the acyl sugars in IL8-1-1 showed an increase in the abundance of several acyl sugars that have one or more C4 acyl chains compared to M82 (Figure 6a,b), including a minor metabolite (S4:14) in which all three C5 fatty acid groups have been replaced by C4. The amounts of acyl sugars without any C4 acyl chains were decreased in IL8-1-1 compared to M82 (Figure 6b).

**Figure 7:**
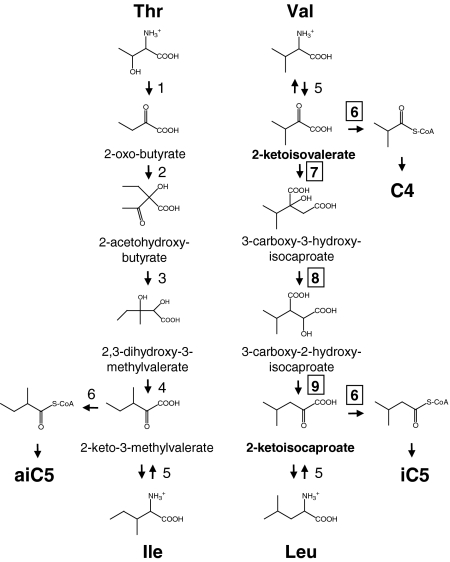
Proposed route to branched acyl chains from branched-chain amino acid precursors. Pathways proposed for the synthesis of precursors to the C4 and C5 fatty acid esters found on acyl sugars. 1, threonine deaminase; 2, acetohydroxyacid synthase; 3, acetohydroxyacid isomeroreductase; 4, dihydroxyacid dehydratase; 5, aminotransferase; 6, branched-chain keto acid dehydrogenase complex; 7, isopropylmalate synthase; 8, isopropylmalate isomerase; 9, isopropylmalate dehydrogenase. Note that steps 7, 8 and 9 are proposed to function in both Leu and iC5 biosynthesis, and a decrease in these activities in ILs 8-1 and 8-1-1 could account for the concomitant reduction of iC5 and increase in C4 compared with M82. Key steps and intermediates described in the text are indicated in bold.

The acyl chains on acyl sugars are proposed to derive from precursors of branched chain amino acid biosynthesis (Kandra *et al.*, 1990; van der Hoeven and Steffens, 2000). Because the C5 acyl chains can derive from precursors of either Leu or Ile, two types of C5 acyl chains can be present. 3-methylbutyrate (iso-C5, abbreviated iC5) comes from the Leu biosynthetic pathway, and 2-methylbutyrate (anteiso-C5, abbreviated aiC5) comes from the Ile pathway (Figure 7).

**Figure 8:**
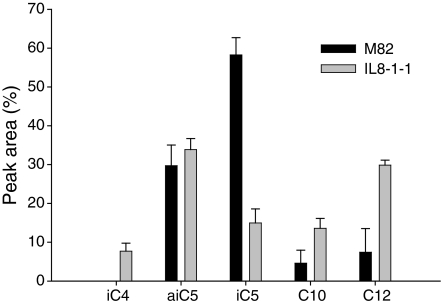
The increase in IL8-1-1 of isobutyrate (C4) is associated with a decrease in 3-methylbutyrate (iC5) side chains. The side chains on acyl sugars from leaf dip samples were trans-esterified to the corresponding ethyl esters and analyzed by GC-MS as described by Gu *et al.* (2010). The peak area percentage values for the peaks integrated from the total ion chromatogram (TIC) for each fatty acid in M82 (*n*=5) and IL8-1-1 (*n*=2) are shown with standard deviation. Despite C4 acyl chain detection in LC-TOF-MS experiments, C4 ethyl ester levels were below the limits of quantification for M82 in this experiment, presumably due to the low abundance and high volatility of this product.

The higher proportion of acyl sugars with C4 acyl chains, the corresponding decrease in C5 acyl chains, and the inability of LC-MS to distinguish branched and linear fatty acid groups prompted us to further characterize the types of acyl chains present on acyl sugars in M82 and IL8-1-1. Acyl chains on the acyl sugars were trans-esterified to the corresponding ethyl esters for analysis by GC-MS. This type of analysis allows identification of the C5 acyl chains as straight chain, iC5 or aiC5. Following ethyl trans-esterification, the leaf dips from IL8-1-1 showed an increase in isobutyrate (C4) ethyl ester compared to M82 (Figure 8). This increase in the C4 chain was associated with a decrease in 3-methylbutyrate ethyl ester (corresponding to iC5), with no decrease in the 2-methylbutyrate ethyl ester (corresponding to aiC5).

### Two ILs with lower total acyl sugars

**Figure 9:**
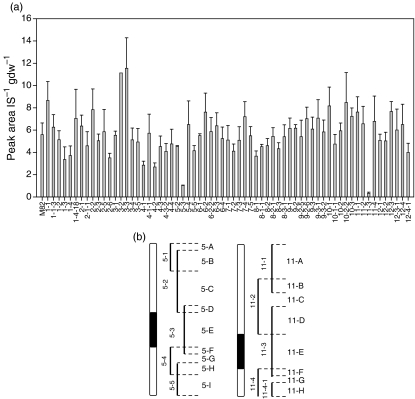
Total acyl sugar levels are decreased in IL5-3 and IL11-3. (a) Total acyl sugar levels were quantified in leaf dips of the ILs by LC-TOF-MS. The sum of the peak areas of detectable acyl sugars was normalized to an internal standard (propyl-4-hydroxybenzoate) and dry leaf weight. Acyl sugars are detected under ESI negative mode. Error bars indicate standard error [*n*=2–4 for the ILs (except IL3-2, *n*=1) and *n*=30 for M82]. (b) Schematic representation of chromosome 5 and 11 introgressions showing loci controlling acyl sugar accumulation on bins 5-E and 11-E.

The results from screening the ILs illustrate that the rapid LC-TOF-MS method revealed quantitative changes in metabolite levels. In contrast to the effect of the *S. pennellii* chromosomal regions 1-3/1-4 and 8-1/8-1-1 on acyl sugar substitution, two other ILs caused changes in total acyl sugar levels. Figure 9(a) shows the results of a screening analysis of total acyl sugars from the chromosome substitution lines, and indicates that ILs 5-3 and 11-3 have consistently lower accumulation of all major acyl sugars compared to M82 (IL5-3, *P*<0.005; IL11-3, *P*<0.001; Mann–Whitney rank sum test). The finding of decreased acyl sugar levels caused by introgression of chromosomal segments from *S. pennellii* LA0716 into M82 tomato is counter-intuitive because this wild tomato species accumulates much higher amounts of acyl sugars than the cultivated tomato (Fobes *et al.*, 1985). Two general hypotheses would account for this unexpected behavior: either there are alleles of M82 that are important for normal acyl sugar levels or there are alleles from LA0716 that, when removed from the LA0716 genomic context, negatively influence acyl sugar accumulation compared to the genes from M82 tomato.

### Discovery of differences in accumulation of metabolites of lower abundance

**Figure 10:**
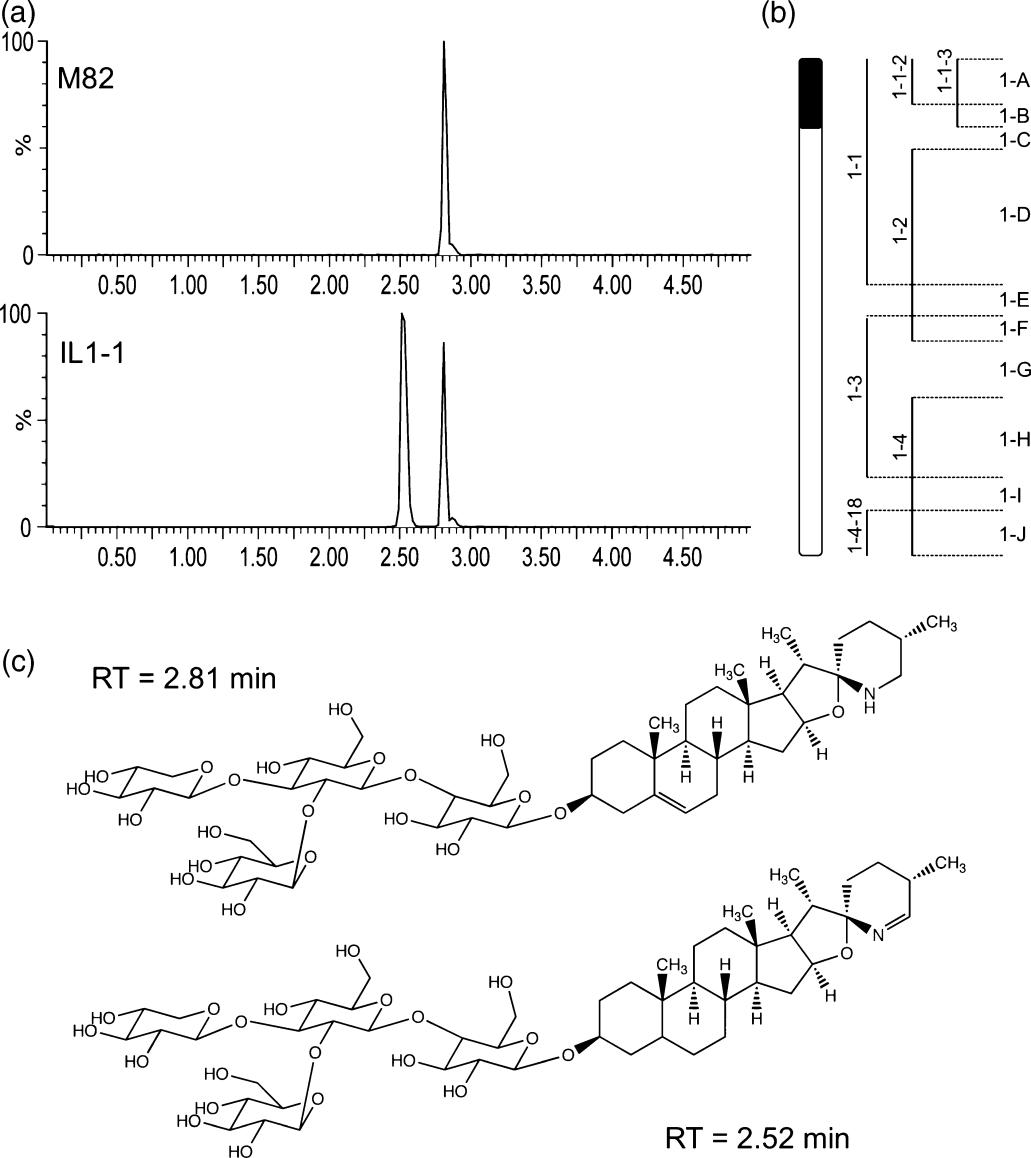
ILs 1-1 (shown) and 1-1-3 accumulate an isomer of dehydrotomatine that is not found in M82. (a) Extracted ion chromatograms of M82 and IL1-1 for *m/z* 1076.5 show an earlier eluting peak found only in the IL and not in M82. (b) Schematic representation of chromosome 1 introgressions showing the locus controlling the glycoalkaloid phenotype on bin 1-A or 1-B (IL1-1-2 was not analyzed in this study). (c) Structures for the dehydrotomatine isomers. The compound with a retention time of 2.81 min and a double bond in the B-ring has been described previously (Friedman *et al.*, 1997). The structure of the compound with a retention time of 2.52 min with the double bond in the F-ring is inferred based on fragmentation from positive-ion mode MS. ‘B’ and ‘F’ refer to the B- and F-rings of the molecule, respectively.

Abundant metabolites that distinguish ILs from a reference genotype can be recognized by visual inspection of LC-MS chromatograms or using statistical methods such as principle component analysis (Figures S4 and S5). In contrast, discovery of changes in the accumulation of less abundant metabolites requires alternative data mining approaches. Low-abundance metabolites are often obscured in principal component analysis plots, and rapid separations increase the likelihood of metabolite co-elution. These challenges can be overcome through automated extraction of metabolite signals and the use of multi-variate statistics to identify metabolites that distinguish genotypes. In the current study, extracted ion chromatograms for the glycoalkaloid dehydrotomatine (*m/z* 1076.5 for the formate adduct ion, negative-ion mode) were generated and integrated, as this dehydrogenated form of tomatine had been found in earlier studies (Friedman *et al.*, 1997; Vaananen *et al.*, 2006). The chromatograms also revealed an earlier-eluting isomer of dehydrotomatine that was present only in samples from the overlapping ILs 1-1 and 1-1-3 and not in M82 (Figure 10). Fragmentation patterns generated using positive-ion mode suggested that the double bond in the early-eluting isomer was located in the F-ring, probably in the form of an imine group (Figure 10c).

Once ILs exhibiting metabolite variation have been identified, deeper probing of differences can be achieved using a supervised multi-variate statistical analysis termed ‘orthogonal projection to latent structures-discriminant analysis’ (OPLS-DA) (Bylesjo *et al.*, 2008). This algorithm employs multiple linear regression and orthogonal filtering to aid in recognition of metabolites that discriminate between sample classes. In this case, the two classes were the M82 parent (class 1) and IL1-1-3 (class 2), and the LC-MS data were automatically extracted using MarkerLynx software (Waters, http://www.waters.com) to yield an array of intensities of analytical signals (ions of specific *m/z* value eluting at a specific time). These processing steps generate lists of peak areas annotated with specific LC retention times and masses; the statistical analysis assigns a p(corr) score suggestive of how well they discriminate classes, and this score is independent of the metabolite abundance. Analysis by OPLS-DA suggested four analytical signals whose abundance is higher in 1-1-3 compared with M82 (Figure S6). These were manually annotated as didehydrotomatine, hydroxytomatine and two adducts of the early-eluting dehydrotomatine isomer, based on fragmentation patterns and accurate mass measurements. None were detected in the other 63 ILs.

## Discussion

Predictive engineering of plant metabolism is an important long-term goal of plant biochemistry (Sweetlove *et al.*, 2003). A key prerequisite is understanding how each of the compounds in a plant cell is synthesized and the mechanisms regulating these biosynthetic pathways. This is an especially daunting task in plants because they produce thousands of compounds, including structurally complex and diverse specialized metabolites (Fridman and Pichersky, 2005; DellaPenna and Last, 2008). New mechanisms and pathways of specialized metabolism continue to be discovered, even for the synthesis of well-characterized metabolites such as terpenes (Sallaud *et al.*, 2009; Schilmiller *et al.*, 2009).

Forward genetics is a well-established approach to tackling uncharacterized biosynthetic pathways. While many analytical methods can be employed for screening genetically diverse germplasms, MS-based methods are especially suited to analysis of multiple compounds within a chemical class (Jander *et al.*, 2004; Gu *et al.*, 2007) or simultaneous monitoring of biosynthetically diverse compounds (Schauer *et al.*, 2006).

Fast and accurate analysis of plant specialized metabolites is especially challenging because of the broad range of structures, chemical properties and concentrations found in any specific tissue, and the lack of availability of standards for most molecules. Typically there are trade-offs between the number of compounds that can be identified, the analytical accuracy of the method, and the speed of the assay and data analysis (Last *et al.*, 2007). We developed an LC-TOF-MS approach that is rapid and provides high-accuracy mass measurements to screen for variants in specialized metabolism without performing derivatization. This technology is broadly accessible, utilizing an ultra-performance fused-core reverse-phase column and standard HPLC equipment coupled with TOF-MS instead of ultra-high-performance LC, ultra-high resolution MS or multi-dimensional MS^*n*^. Narrow-bore capillary GC-MS technology was used for rapid and high efficiency separations of volatile compounds such as terpenes and fatty acid esters.

The genetic screen revealed a variety of changes in specialized metabolites across biosynthetically diverse classes of compounds. Despite relatively low sample replication in screening the germplasm (a maximum of two plants per independent growth cycle and two independent growth cycles), it was possible to identify concomitant reductions in both classes of terpenes, sesquiterpenes alone and all major acyl sugars found in M82 tomato leaf dips. This indicates that the simple extraction method used in this study is quite reproducible. The screen also revealed qualitative changes in the types of monoterpenes, acyl sugars and glycoalkaloids accumulating in specific ILs. It is interesting to note that the trichome metabolites that accumulate in *S. pennellii* LA0716 are very different in quantity and quality from those accumulating in M82 and the ILs. For example, this species produces copious quantities of glucose triester acyl sugars, but M82 lacks detectable levels of this acyl sugar type (Figure S7).

### Qualitative changes in trichome metabolism

Introduction of the IL1-3 and 1-4 overlap region from *S. pennellii* LA0716 leads to production of the monoterpene α-thujene (Figure 3). This monoterpene is present in *S. pennellii* LA0716 leaf trichomes, but is undetectable in M82. The most parsimonious explanation for this phenotype is introduction of an *S. pennellii* monoterpene synthase into the M82 genetic background. The major monoterpenes produced in M82 trichomes are synthesized via the enzyme phellandrene synthase 1, which is encoded by the *PHS1* gene on chromosome 8. Introgression of the *S. pennellii* LA0716 region containing this gene results in ILs 8-1 and 8-1-1, which accumulate *S. pennellii* monoterpenes including α-thujene (Schilmiller *et al.*, 2009). Because separate introduction *S. pennellii* genes on chromosomes 1 (ILs 1-3/1-4) (Figure 3) or 8 (ILs 8-1/8-1-1) (Schilmiller *et al.*, 2009) is sufficient to cause production of α-thujene in M82 tomato, we hypothesize that there are monoterpene synthases capable of producing α-thujene on both chromosomes 1 and 8 of *S. pennellii*.

Changes were also discovered for glycoalkaloid production. ILs 1-1 and 1-1-3, which contain overlapping introgressions, both accumulated an earlier-eluting isomer of dehydrotomatine (Figure 10). We hypothesize that this metabolite could be converted to tomatine by the action of a reductase on the imine double bond and that this activity is deficient in these ILs. The use of OPLS-DA to search for other changes in ILs 1-1 and 1-1-3 also revealed accumulation of compounds tentatively assigned as didehydrotomatine and hydroxytomatine. These findings show that OPLS-DA allows the discovery of changes in compounds of relatively low abundance (<1% of the most abundant compound S4:17).

The biosynthesis of acyl sugars is relatively poorly understood. Feeding studies demonstrated the involvement of branched chain amino acid pathway intermediates as a source of branched acyl chains (Figure 7) (Kandra and Wagner, 1990; Walters and Steffens, 1990). Previously published results are consistent with the hypothesis that these short acyl chains are then elongated to produce the variety of chain lengths observed in tomato acyl sugars (van der Hoeven and Steffens, 2000; Kroumova and Wagner, 2003; Slocombe *et al.*, 2008). However, very little is known about how the acyl sugars are assembled. Enzymes possessing activities consistent with putative early steps in the pathway were identified only for *S. pennellii*, from which two glucosyltransferases were purified based upon the ability to form 1-*O*-acyl-β-glucose from UDP-glucose and a free fatty acid *in vitro* (Ghangas and Steffens, 1993; Kuai *et al.*, 1997). A serine carboxypeptidase-like acyltransferase was identified based on an ability to catalyze the *in vitro* disproportionation of two molecules of 1-*O*-acyl-β-glucose to give diacylglucose and free glucose (Li *et al.*, 1999; Li and Steffens, 2000). Although a *S. lycopersicum* glucosyltransferase activity from trichomes was found to produce the monoester 1-*O*-isobutryl-β-glucose, no acyltransferase activity was detected that produced poly-acylated sugars of the sort found in M82 tomato trichomes (Ghangas and Steffens, 1993). This negative result may reflect the fact that *S. lycopersicum* synthesizes acyl sugars at a dramatically reduced level compared with *S. pennellii* trichomes, and the biosynthetic enzyme activities might be quite low in cultivated tomato trichomes. It is possible that *S. lycopersicum* only accumulates measurable quantities of acyl sucroses, not acyl glucoses, because cultivated tomato trichomes do not produce an acyltransferase activity that uses glucose as a substrate.

Our genetic screen revealed loci from *S. pennellii* LA0716 that influence acyl sugar production in several different ways. ILs 1-3 and 1-4 accumulate primarily tri-acyl sucroses that lack the acetyl group found on tetra-acyl sucroses of M82 (Figure 5). The simplest possible explanations for this phenotype are that an acyltransferase normally active in *S. lycopersicum* is not expressed from this region of *S. pennellii* LA0716 or has a substrate specificity different from the M82 gene product. Trichome-specific ESTs (http://www.trichome.msu.edu and GenBank) and the rapidly expanding tomato genome sequence (http://solgenomics.net/) should prove useful in selection of candidate genes controlling this phenotype.

Acyl sugars in ILs 8-1 and 8-1-1 tend to have one or more C4 acyl chains, while the major acyl sugars in M82 have C5 acyl chains (Figure 6). In addition to the increase in C4 chains, these ILs also have a specific decrease in the iso-C5 moiety but not the anteiso-C5 group (Figure 8). The production of C4 and iC5 chain precursors is proposed to be biosynthetically linked because they are derived from intermediates in the Val and Leu biosynthetic pathways (see Figure 7 for the proposed pathways). In this scheme, 2-ketoisovalerate has three possible routes that it can follow: transamination to directly produce Val (step 5), production of 2-methyl-propionyl CoA (C4) via the action of the branched chain oxo-acid dehydrogenase complex (step 6), or Leu biosynthesis through the sequential action of isopropylmalate synthase, isopropylmalate isomerase, isopropylmalate dehydrogenase and an aminotransferase (steps 7, 8, 9 and 5). Prior to transamination to Leu, 2-ketoisocaproate can serve as a substrate for the branched-chain oxo-acid dehydrogenase complex to produce 3-methyl-butyryl CoA, the iC5 precursor (steps 7, 8, 9 and 6). Our working hypothesis for the alteration in IL8-1 and 8-1-1 phenotype is a reduction in the activity of one or more of the enzymes of Leu biosynthesis (steps 7–9) or a dedicated enzyme activity that evolved from one of these biosynthetic enzymes due to substitution of the *S. pennellii* LA0716 chromosomal segment.

### Quantitative changes in trichome metabolism

Despite use of an experimental design with relatively limited sample replication, specific ILs were found to have quantitative changes in structurally diverse trichome metabolites. In none of these cases were the reductions in trichome metabolite levels associated with changes in the density or gross morphology of specific trichome types or overall trichome density. Thus, these genetic introgressions appear to influence the synthesis or activity of metabolic enzymes rather than resulting from indirect effects due to changes in trichome development.

Three different chromosomal regions from *S. pennellii* LA0716 reduce terpene levels in the ILs, consistent with the low concentrations of both monoterpenes and sesquiterpenes in this wild tomato compared with M82 tomato (Schilmiller *et al.*, 2009). IL2-2 is an example of a line with a simultaneous reduction in both monoterpene and sesquiterpene levels (to approximately 40 and 65% of M82 levels, respectively; Figure 1). Coincident changes in these compound classes are unexpected as they are biosynthetically divergent. Two possibilities are that this locus affects trichome-specific synthesis of C5 precursors or influences secretion or storage of products. The reduced sesquiterpene accumulation in ILs 6-2 and 6-2-2 extends previous work showing that sesquiterpene synthases are located on chromosome 6 in tomato (van der Hoeven *et al.*, 2000; Figure S1). This IL phenotype suggests that chromosome 6 sesquiterpene synthases are both necessary and sufficient for normal accumulation of the detectable trichome sesquiterpenes. In this light, it is interesting that IL10-3 showed a approximately 75–90% decrease in sesquiterpene levels, with no change in monoterpene content (Figure 2). Our working hypothesis for the IL10-3 phenotype is that introgression of one or more other classes of genes controls the level of sesquiterpene production. These could be enzymes responsible for farnesyl diphosphate biosynthesis, regulators of the pathway enzymes, or other as yet unknown players in sesquiterpene synthesis or storage.

ILs 5-3 and 11-3 both have reduced total acyl sugar accumulation compared to M82 (Figure 9), and these differences are seen for molecules of differing structures. This is a counter-intuitive result, as *S. pennellii* LA0716, the source of the introgressed regions, produces massive amounts of acyl sugars, especially acyl glucoses (Fobes *et al.*, 1985). Given the current rudimentary state of knowledge about the biosynthesis of acyl sugars, it is anticipated that map-based gene identification approaches will be needed to find the genes responsible for this phenotype. The rapidly increasing genome sequence resources for tomato (http://solgenomics.net/) and its close relative *Solanum tuberosum* (potato; http://www.potatogenome.net) will expedite gene identification by positional cloning.

The role of acyl sugars as insect feeding deterrents (Goffreda *et al.*, 1989) has led to efforts to breed varieties of cultivated tomatoes that accumulate high levels of acyl sugars (Lawson *et al.*, 1997). QTL were identified from a *S. lycopersicum*×*S. pennellii* cross that had effects on acyl sugar production in terms of the amount accumulated or composition (sugar type or fatty acid types present) (Mutschler *et al.*, 1996). One QTL was located in the same chromosomal region as IL11-3, which has been shown to have an effect on acyl sugars in this study (Figure 9) (Mutschler *et al.*, 1996; Lawson *et al.*, 1997). These quantitative genetics studies demonstrated that specific combinations of multiple unlinked QTL are required to increase acyl sugars in cultivated tomato, making it difficult to create isogenic lines increased in these specialized metabolites. The identification of differences in quantities or types of acyl sugars, terpenes or glycoalkaloids in ILs with single chromosome region substitutions will allow critical analysis of the proposed biological roles for these compounds. In addition to testing the roles of these compounds in mediating interactions with insects and microbes, these lines should enable discovery of the genes and enzymes that control these biosynthetic pathways.

## Experimental procedures

### Plant growth conditions

Tomato seed, *S. lycopersicum* cv. M82 and *S. pennellii* LA0716, was obtained from the Tomato Genetic Resource Center (http://tgrc/ucdavis.edu). *Solanum pennellii* ILs (Eshed and Zamir, 1995) were obtained from Dr Dani Zamir (Hebrew University Faculty of Agriculture, Rehovot, Israel). Plant seedlings were grown in Jiffy peat pots (Hummert International, http://www.hummert.com/) for 3 weeks in a growth chamber maintained for 16 h at 28°C in the light (300 μE m^−2^ sec^−1^, mixed cool white and incandescent light bulbs) and 8 h at 20°C in the dark.

### Sample extraction for GC-MS and LC-MS

The leaflet from the next to youngest leaf of 3-week-old plants were used for chemical analysis of volatile and non-volatile metabolites. For extraction of volatile compounds, a leaflet was placed in a 1.5 ml microcentrifuge tube containing 750 μl of *tert*-butyl methyl ether (MTBE) with 10 ng μl^−1^ of tetradecane internal standard and gently rocked for 1 min. To extract non-volatile compounds, a leaflet was dipped in 1 ml of isopropanol:acetonitrile:water (3:3:2 v/v/v) containing 0.1% formic acid and 10 μm of propyl-4-hydroxybenzoate as internal standard, with gentle rocking for 1 min as described above.

Individual sets of ILs plus a total of 30 M82 control plants (14 and 16 plants in the first and second growth cycles, respectively) were grown independently at different times in the same growth chamber under the growth conditions mentioned above and harvested for chemical analysis. Of the 76 ILs developed to cover the entire genome of the recurrent parent M82, some lines failed to germinate, resulting in screening of 65 ILs. Only one small region of the genome on the top of chromosome 5 (bin 5A unique to IL5-1) was not assayed. A total of three or four samples were tested for each IL that was grown, except for IL3-2 (*n*=1) and IL2-6 (*n*=2).

### GC-MS and LC-MS methods

GC-MS was performed to analyze the profile of volatile compounds using a 6890N network GC system with 5975B inert XL MSD detector (Agilent Technologies, http://www.agilent.com). Separation was achieved by injection of 1 μl of extract into a 127-501N DB-5 column (10 m × 0.1 mm × 0.34 μm; Agilent) using the following temperature profile: 40°C for 1 min; 30°C min^−1^ to 90°C; 5°C min^−1^ to 110°C; 40°C min^−1^ to 165°C; 5°C min^−1^ to 180°C; 40°C min^−1^ to 320°C; 320°C for 2 min. Volatile metabolites were identified by comparing their *m/z* values with the ChemStation database (Agilent Technologies), and peak areas were integrated using QuanLynx software (Waters). The amount of compounds was normalized to the tetradecane internal standard and dry leaf weight, and quantified using a standard curve of γ-terpinene as the external standard.

LC-MS (LC-20AD, Shimadzu, http://www.shimadzu.com; LCT Premier, Waters) was used for the analysis of non-volatile metabolites by modifications of a method recently described for glucosinolate analysis (Gu *et al.*, 2010). Details are given in Appendix S1.

## SUPPORTING INFORMATION

**Figure S1.** ILs 6-2 and 6-2-2 do not produce detectable levels of sesquiterpenes. The overlapping introgressions on chromosome 6 (IL6-2 and IL6-2-2) contain the loci responsible for controlling sesquiterpene accumulation in *S. lycopersicum*. ILs 6-2 and 6-2-2 show the *S*. *pennellii* LA0716 phenotype, which does not accumulate sesquiterpenes in leaf trichomes. Shown are extracted ion chromatograms for *m/z* 93, a fragment ion common in terpenes. 1, α-pinene; 2, δ-carene; 3, α-phellandrene; 4, α-terpinene; 5, limonene + β-phellandrene; 6, δ-elemene; 7, caryophyllene; 8, α-humulene.

**Figure S2.** Extracted ion chromatograms for leaf dip samples of IL8-1-1. Extracted ion chromatograms show peaks corresponding to tri- and tetraacylsucroses ranging from *m/z* 639 to *m/z* 779 differing by 14 Da in molecular mass owing to additional methylene groups in the fatty acid moieties. Left: 43 min gradient, Right: 5 min gradient; with negative mode ESI TOF MS. Acylsucroses having identical nominal mass but are separated chromatographically and distinguished by accurate mass measurements, correspond to a tetraacylsucrose and a triacylsucrose (i.e., S4:21 and S3:22). Additional evidence allowing these assignments to be made comes from high mass accuracy measurements that allow resolution of the 36.4 milliDalton difference in molecular masses between the triacylated and tetraacylated compounds.

**Figure S3.** NMR data reveal chemical structure of tetraacylsucrose S4:17 [2-*O*-acetyl, 3-*O*-(2-methylbutanoyl), *O*-4,3’-bis(3-methylbutanoyl)sucrose]. This compound was isolated from extracts of *S. lycopersicum* M82 and ^1^H and ^13^C NMR resonance assignments are provided. NMR assignments are based on a composite of information from ^1^H, ^13^C, DEPT, gHMQC, gHMBC, gCOSY, and TOCSY spectra. The sample extract contained approximately 30% of a related isomer. ^a^Signals may be interchanged. ^b^Spectral overlap prohibits definitive assignment.

**Figure S4.** Principal component analysis (PCA) of LC-TOF MS metabolite data.

**Figure S5.** Statistical analysis of selected IL data.

**Figure S6.** Glycoalkaloid differences are detected in IL1-1-3 compared to M82 using Orthogonal Projection to Latent Structures-Discriminant Analysis (OPLS-DA) of measured metabolite signals. OPLS-DA loading ‘S-plot’ represents the interclass difference between IL1-1-3 and M82 is primarily attributed to analytical signals corresponding to *m/z* 1066 (‘A’, chloride adduct of the early eluting isomer of dehydrotomatine), 1074 (‘B’ formate adduct of didehydrotomatine), 1076 (‘C’, formate adduct of early eluting isomer of dehydrotomatine), and 1094 (‘D’, formate adduct of putative hydroxytomatine). Results for IL1-1 are similar to those shown here for IL1-1-3. Relative LC/MS signals for the highlighted metabolites can be compared to the signal of the most abundant metabolite signal (tetraacylsucrose S4:17). Signals for the highlighted glycoalkaloids ranged from 0.8% (compound ‘B’) to 18% (compound ‘C’) relative to the signal for S4:17.

**Figure S7.** Comparison of LC-TOF total ion chromatograms reveals large differences in abundance and specific metabolites in the parental lines M82 and LA0716. Some abundant acyl sugars are indicated (G for glucose, S for sucrose) to highlight differences. Chromatograms are from leaf dips and response sensitivities are set the same to show differences in peak areas.

**Table S1.** MarkerLynx marker report of the full data set generated screening the S. lycopersicum M82 X S. pennellii LA0716 ILs using LC-TOF MS. Data are reported for all samples successfully run and analyzed. Note that only the M82 tomato S4:17 structure was confirmed by NMR and all other metabolite annotations are our best estimates and need to be confirmed by other methods. Other analytical signals have not been assigned putative compound structures or classes.

**Appendix S1.** Supplementary methods.

Please note: As a service to our authors and readers, this journal provides supporting information supplied by the authors. Such materials are peer-reviewed and may be re-organized for online delivery, but are not copy-edited or typeset. Technical support issues arising from supporting information (other than missing files) should be addressed to the authors.

## References

[b1] Allen J, Davey HM, Broadhurst D, Heald JK, Rowland JJ, Oliver SG, Kell DB (2003). High-throughput classification of yeast mutants for functional genomics using metabolic footprinting. Nat. Biotechnol..

[b2] Benning C (2004). Genetic mutant screening by direct metabolite analysis. Anal. Biochem..

[b3] Browse J, McCourt P, Somerville CR (1985). A mutant of Arabidopsis lacking a chloroplast-specific lipid. Science.

[b4] Bylesjo M, Rantalainen M, Nicholson JK, Holmes E, Trygg J (2008). K-OPLS package: kernel-based orthogonal projections to latent structures for prediction and interpretation in feature space. BMC Bioinformatics.

[b5] Castrillo JI, Hayes A, Mohammed S, Gaskell SJ, Oliver SG (2003). An optimized protocol for metabolome analysis in yeast using direct infusion electrospray mass spectrometry. Phytochemistry.

[b6] Croteau RB, Davis EM, Ringer KL, Wildung MR (2005). (-)-Menthol biosynthesis and molecular genetics. Naturwissenschaften.

[b7] Cunliffe JM, Adams-Hall SB, Maloney TD (2007). Evaluation and comparison of very high pressure liquid chromatography systems for the separation and validation of pharmaceutical compounds. J. Sep. Sci..

[b8] DellaPenna D, Last RL (2008). Genome-enabled approaches shed new light on plant metabolism. Science.

[b9] Dettmer K, Aronov PA, Hammock BD (2007). Mass spectrometry-based metabolomics. Mass Spectrom. Rev..

[b10] Duke MV, Paul RN, Elsohly HN, Sturtz G, Duke SO (1994). Localization of artemisinin and artemisitene in foliar tissues of glanded and glandless biotypes of *Artemisia annua* L. Int. J. Plant Sci..

[b11] Eshed Y, Zamir D (1994). A genomic library of *Lycopersicon pennellii* in *L. esculentum*: a tool for fine mapping of genes. Euphytica.

[b12] Eshed Y, Zamir D (1995). An introgression line population of *Lycopersicon pennellii* in the cultivated tomato enables the identification and fine mapping of yield-associated QTL. Genetics.

[b13] Fobes JF, Mudd JB, Marsden MP (1985). Epicuticular lipid accumulation on the leaves of *Lycopersicon pennellii* (Corr.) D’Arcy and *Lycopersicon esculentum* Mill. Plant Physiol..

[b14] Fridman E, Pichersky E (2005). Metabolomics, genomics, proteomics, and the identification of enzymes and their substrates and products. Curr. Opin. Plant Biol..

[b15] Friedman M, Kozukue N, Harden LA (1997). Structure of the tomato glycoalkaloid tomatidenol-3-β-lycotetraose (dehydrotomatine). J. Agric. Food. Chem..

[b16] Galpaz N, Ronen G, Khalfa Z, Zamir D, Hirschberg J (2006). A chromoplast-specific carotenoid biosynthesis pathway is revealed by cloning of the tomato white-flower locus. Plant Cell.

[b17] Gang DR, Wang J, Dudareva N, Nam KH, Simon JE, Lewinsohn E, Pichersky E (2001). An investigation of the storage and biosynthesis of phenylpropenes in sweet basil. Plant Physiol..

[b18] Ghangas GS, Steffens JC (1993). UDPglucose: fatty acid transglucosylation and transacylation in triacylglucose biosynthesis. Proc. Natl Acad. Sci. USA.

[b19] Goffreda JC, Mutschler MA, Ave DA, Tingey WM, Steffens JC (1989). Aphid deterrence by glucose esters in glandular trichome exudate of the wild tomato *Lycopersicon pennellii*. J. Chem. Ecol..

[b20] Goodacre R, York EV, Heald JK, Scott IM (2003). Chemometric discrimination of unfractionated plant extracts analyzed by electrospray mass spectrometry. Phytochemistry.

[b21] Gu L, Jones AD, Last RL (2007). LC-MS/MS assay for protein amino acids and metabolically related compounds for large-scale screening of metabolic phenotypes. Anal. Chem..

[b22] Gu L, Jones AD, Last RL (2010). Broad connections in the Arabidopsis seed metabolic network revealed by metabolite profiling of an amino acid catabolism mutant. Plant J..

[b23] van der Hoeven RS, Steffens JC (2000). Biosynthesis and elongation of short- and medium-chain-length fatty acids. Plant Physiol..

[b24] van der Hoeven RS, Monforte AJ, Breeden D, Tanksley SD, Steffens JC (2000). Genetic control and evolution of sesquiterpene biosynthesis in *Lycopersicon esculentum* and *L. hirsutum*. Plant Cell.

[b25] Hsieh Y, Duncan CJ, Brisson JM (2007). Fused-core silica column high-performance liquid chromatography/tandem mass spectrometric determination of rimonabant in mouse plasma. Anal. Chem..

[b26] Iijima Y, Davidovich-Rikanati R, Fridman E, Gang DR, Bar E, Lewinsohn E, Pichersky E (2004). The biochemical and molecular basis for the divergent patterns in the biosynthesis of terpenes and phenylpropenes in the peltate glands of three cultivars of basil. Plant Physiol..

[b27] Isaacson T, Ronen G, Zamir D, Hirschberg J (2002). Cloning of tangerine from tomato reveals a carotenoid isomerase essential for the production of beta-carotene and xanthophylls in plants. Plant Cell.

[b28] Jander G, Norris SR, Joshi V, Fraga M, Rugg A, Yu S, Li L, Last RL (2004). Application of a high-throughput HPLC-MS/MS assay to Arabidopsis mutant screening; evidence that threonine aldolase plays a role in seed nutritional quality. Plant J..

[b29] Kandra L, Wagner GJ (1990). Chlorsulfuron modifies biosynthesis of acyl acid substituents of sucrose esters secreted by tobacco trichomes. Plant Physiol..

[b30] Kandra G, Severson R, Wagner GJ (1990). Modified branched-chain amino acid pathways give rise to acyl acids of sucrose esters exuded from tobacco leaf trichomes. Eur. J. Biochem..

[b31] Kirkland JJ, Langlois TJ, DeStefano JJ (2007). Fused core particles for HPLC columns. Am. Lab..

[b32] Kliebenstein DJ, D’Auria JC, Behere AS, Kim JH, Gunderson KL, Breen JN, Lee G, Gershenzon J, Last RL, Jander G (2007). Characterization of seed-specific benzoyloxyglucosinolate mutations in *Arabidopsis thaliana*. Plant J..

[b33] Kroumova AB, Wagner GJ (2003). Different elongation pathways in the biosynthesis of acyl groups of trichome exudate sugar esters from various solanaceous plants. Planta.

[b34] Kuai JP, Ghangas GS, Steffens JC (1997). Regulation of triacylglucose fatty acid composition (uridine diphosphate glucose: fatty acid glucosyltransferases with overlapping chain-length specificity). Plant Physiol..

[b35] Last RL, Jones AD, Shachar-Hill Y (2007). Towards the plant metabolome and beyond. Nat. Rev. Mol. Cell Biol..

[b36] Lawson DM, Lunde CF, Mutschler MA (1997). Marker-assisted transfer of acyl sugar-mediated pest resistance from the wild tomato, *Lycopersicon pennellii*, to the cultivated tomato, *Lycopersicon esculentum*. Mol. Breeding.

[b37] Li AX, Steffens JC (2000). An acyltransferase catalyzing the formation of diacylglucose is a serine carboxypeptidase-like protein. Proc. Natl Acad. Sci. USA.

[b38] Li AX, Eannetta N, Ghangas GS, Steffens JC (1999). Glucose polyester biosynthesis. Purification and characterization of a glucose acyltransferase. Plant Physiol..

[b39] Lu Y, Savage LJ, Ajjawi I (2008). New connections across pathways and cellular processes: industrialized mutant screening reveals novel associations between diverse phenotypes in Arabidopsis. Plant Physiol..

[b40] Mutschler MA, Doerge RW, Liu SC, Kuai JP, Liedl BE, Shapiro JA (1996). QTL analysis of pest resistance in the wild tomato *Lycopersicon pennellii*: QTLs controlling acylsugar level and composition. Theor. Appl. Genet..

[b41] Nagel J, Culley LK, Lu Y, Liu E, Matthews PD, Stevens JF, Page JE (2008). EST analysis of hop glandular trichomes identifies an *O*-methyltransferase that catalyzes the biosynthesis of xanthohumol. Plant Cell.

[b42] Ronen G, Carmel-Goren L, Zamir D, Hirschberg J (2000). An alternative pathway to β-carotene formation in plant chromoplasts discovered by map-based cloning of *Beta* and *old-gold* color mutations in tomato. Proc. Natl Acad. Sci. USA.

[b43] Rounsley SD, Last RL (2010). Shotguns and SNPs: how fast and cheap sequencing is revolutionizing plant biology. Plant J..

[b44] Salisbury JJ (2008). Fused-core particles: a practical alternative to sub-2 micron particles. J. Chromatogr. Sci..

[b45] Sallaud C, Rontein D, Onillon S (2009). A novel pathway for sesquiterpene biosynthesis from *Z*,*Z-*farnesyl pyrophosphate in the wild tomato *Solanum habrochaites*. Plant Cell.

[b46] Sattler SE, Cahoon EB, Coughlan SJ, DellaPenna D (2003). Characterization of tocopherol cyclases from higher plants and cyanobacteria. Evolutionary implications for tocopherol synthesis and function. Plant Physiol..

[b47] Schauer N, Semel Y, Roessner U (2006). Comprehensive metabolic profiling and phenotyping of interspecific introgression lines for tomato improvement. Nat. Biotechnol..

[b48] Schilmiller AL, Last RL, Pichersky E (2008). Harnessing plant trichome biochemistry for the production of useful compounds. Plant J..

[b49] Schilmiller AL, Schauvinhold I, Larson M, Xu R, Charbonneau AL, Schmidt A, Wilkerson C, Last RL, Pichersky E (2009). Monoterpenes in the glandular trichomes of tomato are synthesized from a neryl diphosphate precursor rather than geranyl diphosphate. Proc. Natl Acad. Sci. USA.

[b50] Sirikantaramas S, Taura F, Tanaka Y, Ishikawa Y, Morimoto S, Shoyama Y (2005). Tetrahydrocannabinolic acid synthase, the enzyme controlling marijuana psychoactivity, is secreted into the storage cavity of the glandular trichomes. Plant Cell Physiol..

[b51] Slocombe SP, Schauvinhold I, McQuinn RP (2008). Transcriptomic and reverse genetic analyses of branched-chain fatty acid and acyl sugar production in *Solanum pennellii* and *Nicotiana benthamiana*. Plant Physiol..

[b52] Smedsgaard J (1997). Micro-scale extraction procedure for standardized screening of fungal metabolite production in cultures. J. Chromatogr. A.

[b53] Sweetlove LJ, Last RL, Fernie AR (2003). Predictive metabolic engineering: a goal for systems biology. Plant Physiol..

[b54] Vaananen T, Ikonen T, Rokka V-M, Kuronen P, Serimaa R, Ollilainen V (2006). Influence of incorporated wild Solanum genomes on potato properties in terms of starch nanostructure and glycoalkaloid content. J. Agric. Food. Chem..

[b55] Valentin HE, Lincoln K, Moshiri F (2006). The Arabidopsis *vitamin E pathway gene5-1* mutant reveals a critical role for phytol kinase in seed tocopherol biosynthesis. Plant Cell.

[b56] Wagner GJ (1991). Secreting glandular trichomes: more than just hairs. Plant Physiol..

[b57] Walters DS, Steffens JC (1990). Branched chain amino acid metabolism in the biosynthesis of *Lycopersicon pennellii* glucose esters. Plant Physiol..

[b58] Wilson ID, Nicholson JK, Castro-Perez J, Granger JH, Johnson KA, Smith BW, Plumb RS (2005). High resolution ‘ultra performance’ liquid chromatography coupled to oa-TOF mass spectrometry as a tool for differential metabolic pathway profiling in functional genomic studies. J. Proteome Res..

