# Supplementary material for: Mass spectrometry screening reveals widespread diversity in trichome specialized metabolites of tomato chromosomal substitution lines
Source: Plant J. 2010 Mar 8;62(3):391–403. doi: 10.1111/j.1365-313X.2010.04154.x (PMC2881305; doi:10.1111/j.1365-313X.2010.04154.x)
Supplement: Supplementary file 1 [file tpj0062-0391-SD1.pdf]

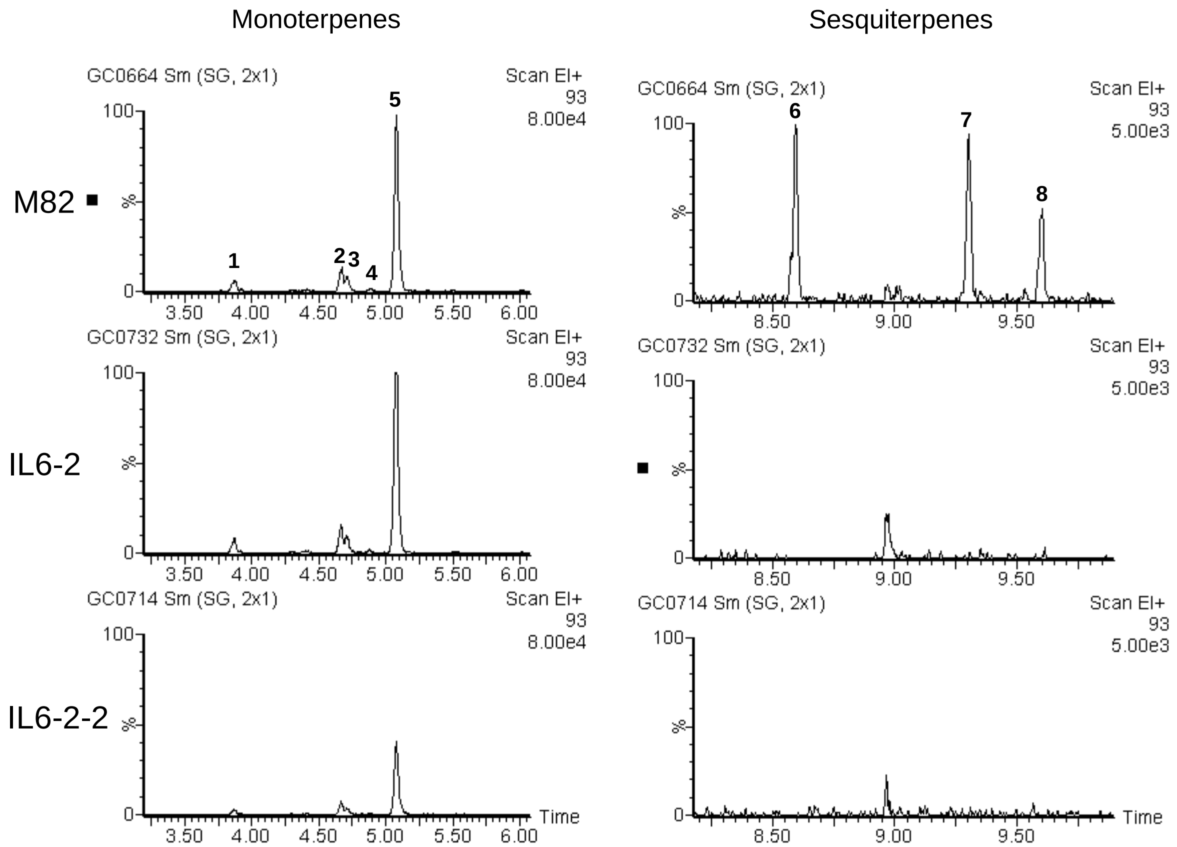

Figure S1. Schilmiller, Shi et al.

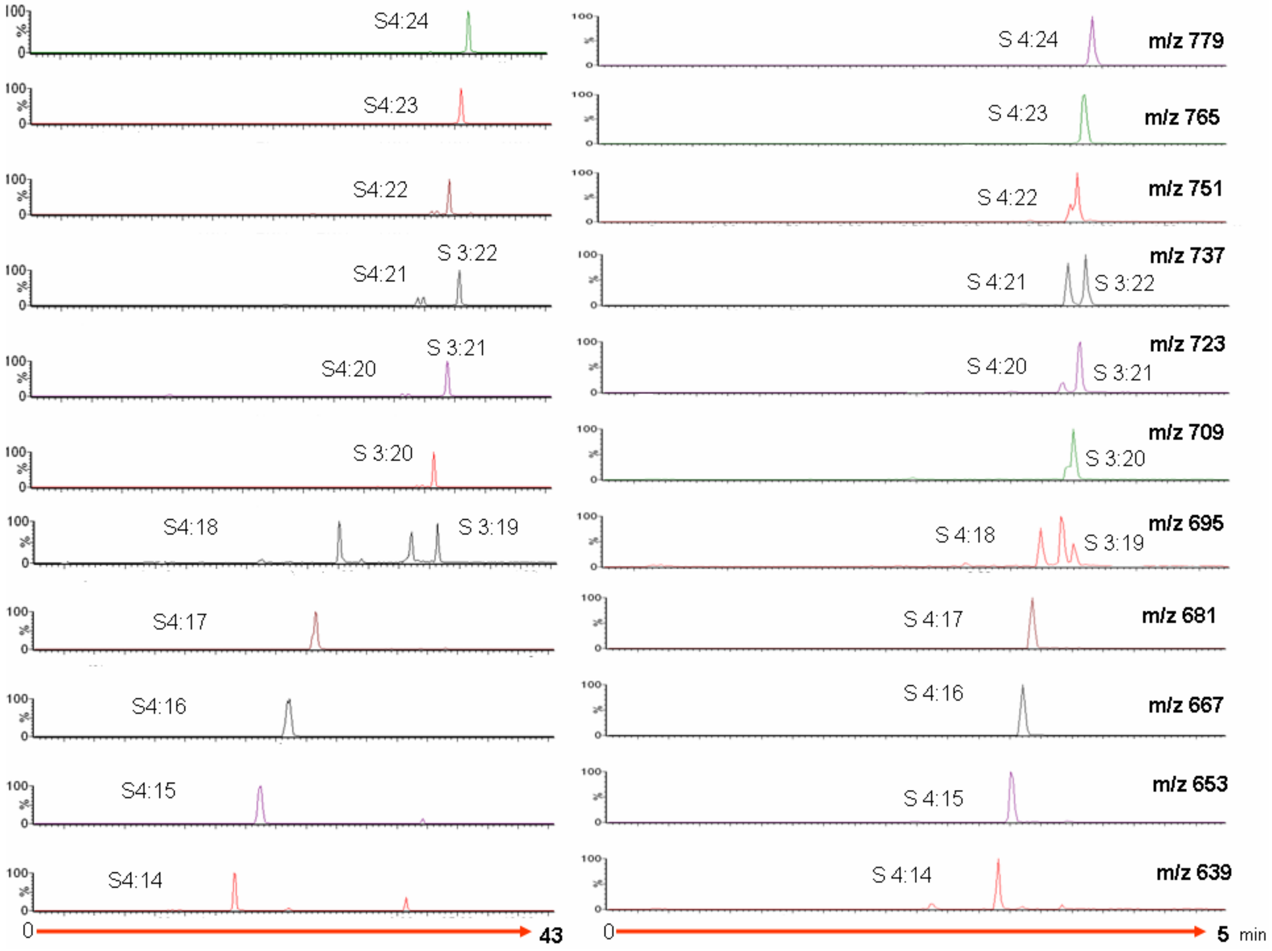

Figure S2. Schilmiller, Shi et al.

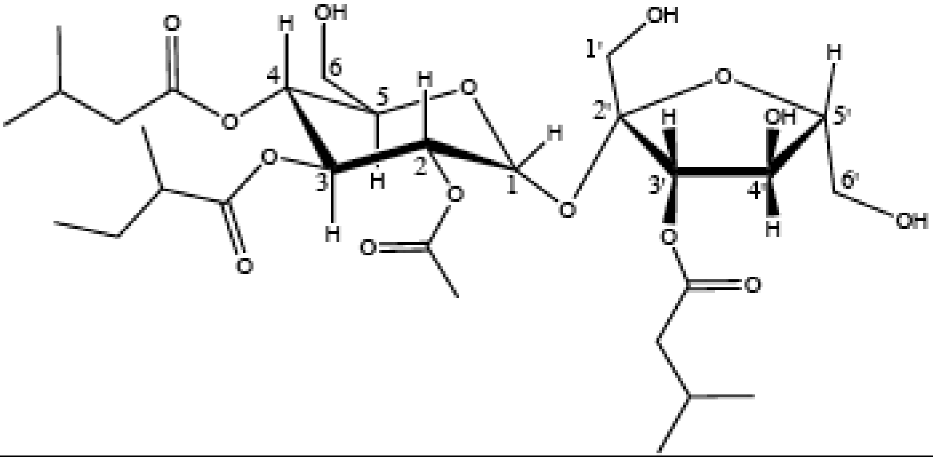

Carbon # (multiplicity)	[1]H *mult.* (*J*, Hz)	[13]C
1 (CH)	5.61 *d* (3.7)	89.3
2 (CH)	4.85 *dd* (10.6, 3.7)	70.5
2-O-		
-1 (CO)	----	170.2
-2 (CH_3)	1.98 *s*	20.6
3 (CH)	5.44 *dd* (10.6, 10.0)	68.9
3-O-		
-1 (CO)	----	175.4
-2 (CH)	2.29 *m*	40.9
-2 (-CH_3)	1.03 *d* (7.0)	16.4
-3 (CH_2)	1.56, 1.37 *sext* (7.0)	26.5
-4 (CH_3)	0.81 *t* (7.4)	11.4
4 (CH)	4.92 *dd* (10.2, 10.0)	68.5
4-O-		
-1 (CO)	----	172.2
-2 (CH_2)	2.1 - 2.4 *m* [b]	42.9
-3 (CH)	2.15 *m*	25.8
-4 (CH_3)	0.9 *d* (7)[a]	22.3
-5 (CH_3)	1.0 *d* (7)[a]	22.3
5 (CH)	4.09 *m*	71.5
6 (CH_2)	3.56, 3.60 *m*	61.4
1′ (CH_2)	3.47, 3.58 *d* (12.3)	64.2
2′ (C)	----	103.9
3′ (CH)	5.21 *d* (7.9)	78.7
3′-O-		
-1 (CO)	----	174.0
-2 (CH_2)	2.3-2.4 *m* [b]	43.0
-3 (CH)	2.03 *m*	25.3
-4 (CH_3)	0.91 *d* (7)[a]	22.3
-5 (CH_3)	1.01 *d* (7)[a]	22.3
4′ (CH)	4.45 *dd* (8.0, 8.0)	71.5
5′ (CH)	3.91 *m*	82.5
6′ (CH_2)	3.70, 3.85 *m*	60.3

Figure S3. Schilmiller, Shi et al.

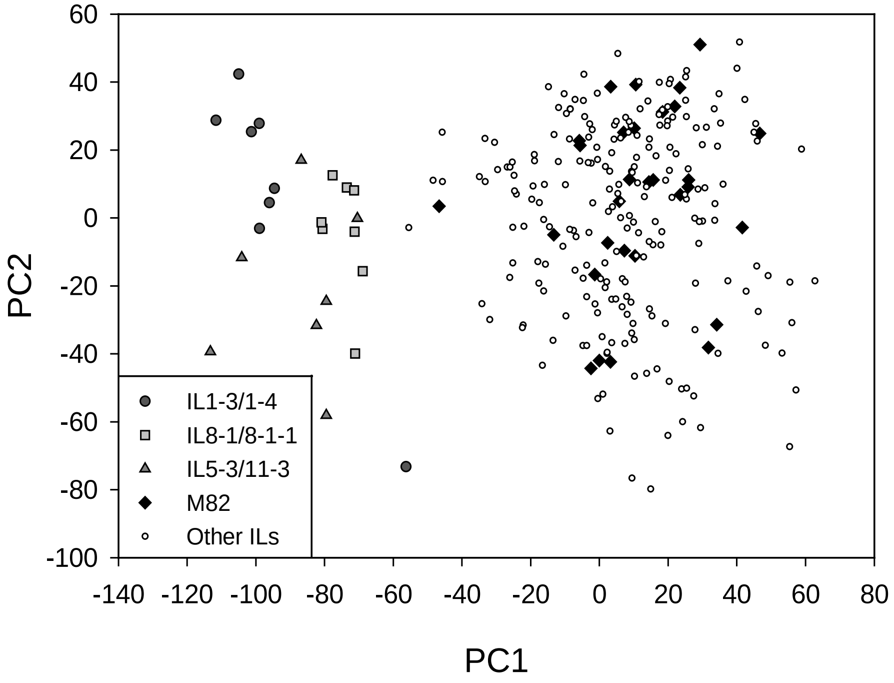

Figure S4 Schilmiller, Shi et al.

A

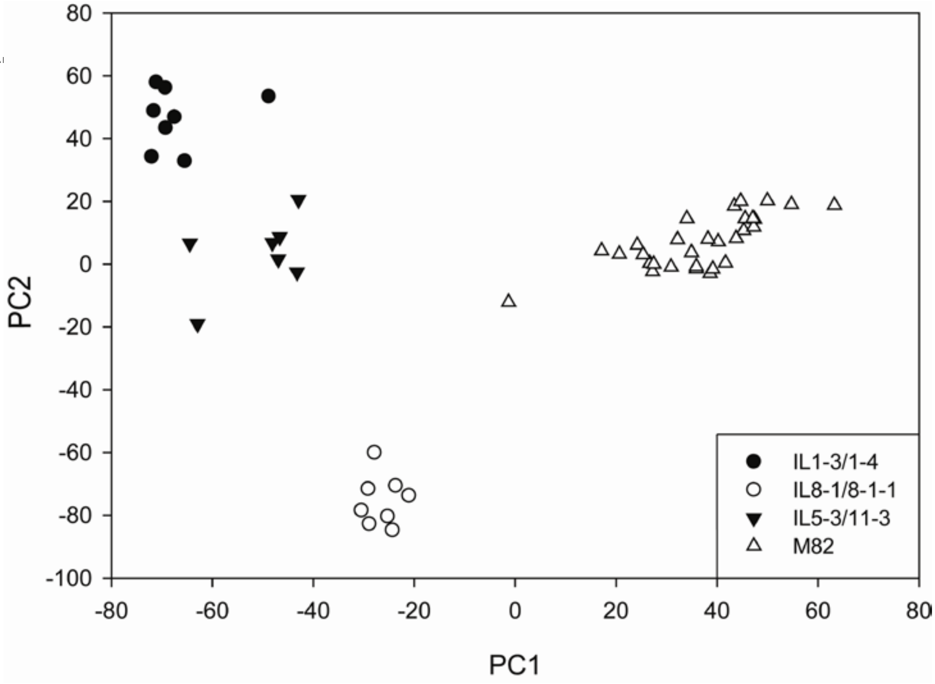

B

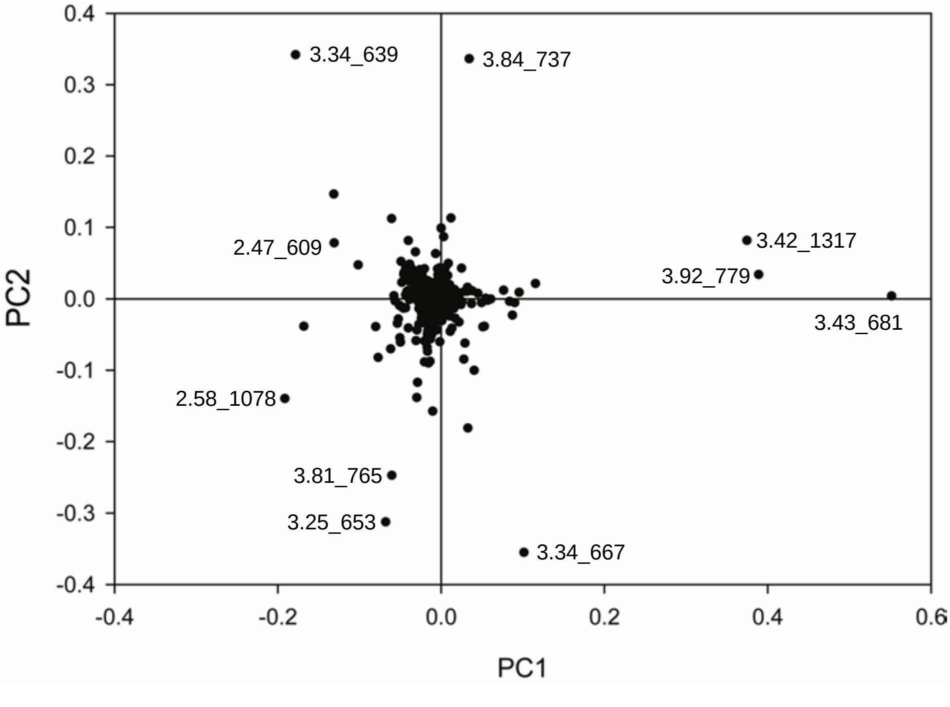

Figure S5 Schilmiller, Shi et al.

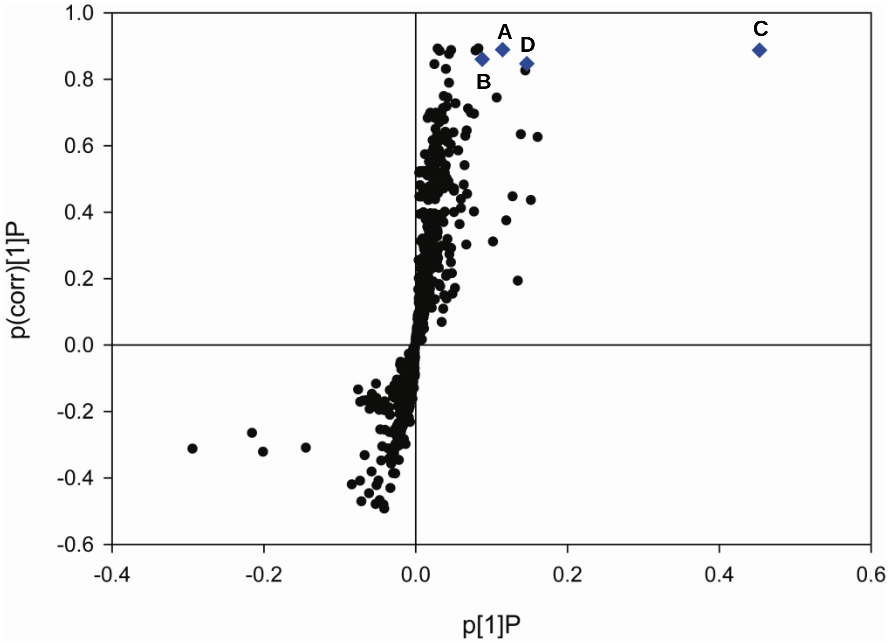

	Retention time_m/z	p[1]P	p(corr)[1]P
A	2.51_1066	0.114	0.889
B	2.47_1074	0.088	0.860
C	2.49_1076	0.453	0.887
D	2.60_1094	0.146	0.847

Figure S6. Schilmiller, Shi et al.

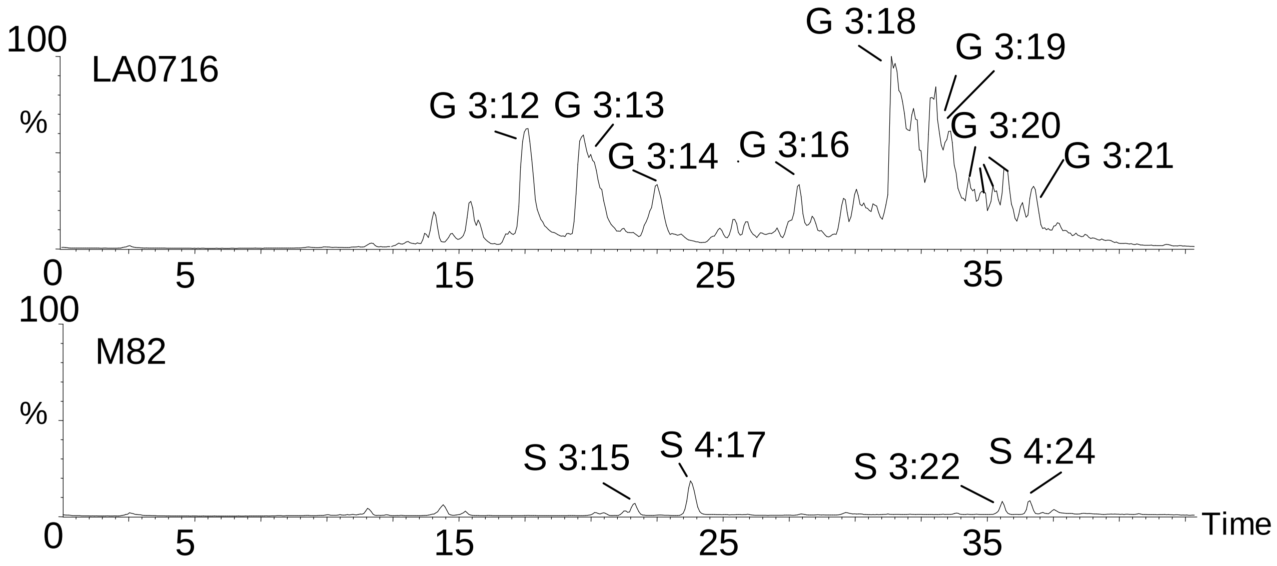

Figure S7. Schilmiller, Shi et al.
