# Supplementary material for: Mass spectrometry screening reveals widespread diversity in trichome specialized metabolites of tomato chromosomal substitution lines
Source: Plant J. 2010 Mar 8;62(3):391–403. doi: 10.1111/j.1365-313X.2010.04154.x (PMC2881305; doi:10.1111/j.1365-313X.2010.04154.x)
Supplement: Supplementary file 3 [file tpj0062-0391-SD3.doc]

**Appendix S1**

**Detailed LC/MS method**

Extracts (10 μl) were injected into a fused core Ascentis Express C18 column (2.1 mm × 50 mm with 2.7 μm particles, Sigma-Aldrich) on a HPLC for reverse-phase analysis. Column temperature was maintained at 30°C. A steep gradient was executed between solvent A and B (A - 0.15% formic acid in MilliQ water, B - methanol) for the analysis time of 5 min/sample with 0.4 ml/min flow rate. Gradient profile was as follows: 10% B for initial step; linear gradient to 60% B to 2 min; 100% B to 3 min; 100% B maintained to 4 min; 10% B to 5 min. MS was coupled to HPLC to identify and detect analytic signals under the conditions as described: electrospray negative ionization mode; 3.00 kV capillary voltage; 90°C source temperature; 300°C desolvation temperature; 20 L/h nebulizer nitrogen flow rate; 300 L/h desolvation nitrogen gas flow rate; mass range of *m/z* 50-1500 Da, with spectra accumulated at 0.1s/function for the 5 min. gradient and 0.4 s/function for the 43-min gradient. Aperture 1 voltage was rapidly switched among five different conditions (10, 25, 40, 55 and 80 V) in the ion transit region of mass spectrometer to generate spectra of specific analyte under non-fragmentation and fragmentation environment, providing structural information as well as molecular ion for the analyte from a single analysis.

Data were acquired in centroid format with dynamic range enhancement (DRE) that enabled a wide dynamic range of quantification. Extracted ion chromatograms were integrated for *m/z* values selected for each selected metabolite and for the internal standard using QuanLynx software.  Metabolite peak areas were divided by the peak area for the internal standard, and the resulting values were normalized to dry leaf weights. Raw data obtained from LC/MS were also analyzed by MarkerLynx software (Waters, applications manager version 4.1) for statistical tests such as principle component analysis (PCA). Parameters for analytes were set as follows: retention time (RT) range 0-5 min; mass range of *m/z* 50-1500 Da; mass tolerance 20 ppm. Internal standard detection parameters were deselected for peak RT alignment, and isotopic peaks were excluded for the analysis. Noise elimination level was 50, and minimal intensity was 1% of base peak intensity. Also, maximum mass per RT was 50, and RT tolerance was 0.2 min. No specific mass or adduct ions were excluded. Ions from different samples were considered same when they demonstrated same RT and *m/z* value with tolerance mentioned above. If peak was not detected in the sample, ion intensity was documented as zero in the final data table. A list of peak intensity was generated for all the samples, using RT and *m/z* pairs as identifiers for each peak. The resulting three types of data including peak number (RT-*m/z* pair), sample name and ion intensity were subjected to PCA. In addition, orthogonal projection to latent structures-discriminant analysis (OPLS-DA) was carried out by SIMCA-P software (Umetrics).

**Analysis of chromosome substitution lines by LC-TOF MS**
Discovery of differences in specific metabolites or total amounts of a class of metabolites required a more complex and automated approach to data analysis owing to the large number of analytical signals generated in this method as compared to the far more limited number of volatile molecules analyzed by GC/MS (see above; note that 'analytical signal' refers to an ion of specific *m/z* value eluting at a specific time). Raw data from the LC/MS were first analyzed by automated peak detection, integration, and alignment and the results were assembled into an extensive data matrix of analytical signals for the set of ILs. Principal components analysis was performed on this data matrix, and results for the first and second components are displayed in Figure S4. While the majority of lines clustered with the M82 samples (shown as diamonds in Figure S4), extracts from six lines were distinguishable as outlier samples. Two pairs were from lines with overlapping chromosomal regions (1-3 plus 1-4 and 8-1 plus 8-1-1), while 5-3 and 11-3 each represents the impact of different chromosomal regions. Thus, the PCA showed that four regions influence the composition of specialized metabolites extracted from the trichomes and surface of the leaves of M82 tomato. Note that a large number of analytical signals whose structures are not currently identified are also found in these studies. Our analysis has focused on classes of compounds for which genetic variation is detected in the ILs.

To understand which classes of metabolites had the strongest impact on separation of these six lines from the M82 parent, the scores plot from PCA of this subset of lines was examined (Figure S5A). This analysis confirmed the separation of the six lines into three groups separate from the M82 recurrent parent. PCA loadings were examined to determine which LC-TOF MS analytical signals contributed to the differences among clusters in the PCA scores plot (Figure S5B). This analysis indicated that the ILs separated from M82 primarily due to differences in the analytical signals with *m/z* 681, 779, and 1317 (tetraacylsucroses). ILs 1-3 and 1-4 were distinguished from the other samples in large part due to differences in analytical signals of *m/z* 639 and 737 (triacylsucroses) while ILs 8-1 and 8-1-1 were altered in *m/z* 653, 667 and 765 (a different set of tetraacylsucroses). In contrast, ILs 5-3 and 11-3 were found to have changes in the proportions of acylsugars relative to tomatine (*m/z* 1078) and rutin (*m/z* 609).

**Extraction and isolation method for purification of S4:17 acylsugar**

Sixty grams of freshly harvested leaves from 3-week old *S. lycopersicum* cv. M82 were extracted by dipping leaf material into 1.0 liter of isopropanol: acetonitrile: water (3:3:2, v/v/v) containing 0.1% formic acid for 5 min. The resulting extract was partitioned into two phases by addition of 500 ml CHCl_3_. The lower chloroform layer was collected and evaporated to dryness on a rotary evaporator. The residue was dissolved in 10 ml Methanol:H_2_O (1:1, v/v) for further purification using a Supelclean^TM^ LC-C18 solid phase extraction (SPE) (10 ml column). A 0.2 ml aliquot was loaded onto the SPE column for each step of the process, and the process was repeated 25 times to accumulate sufficient quantity for NMR analysis. Material was eluted using mixtures of methanol/water, using 0.1% formic acid in the aqueous component for the following methanol/aqueous compositions: Fraction 1 = 20 ml of Methanol:H_2_O (9:11,v/v); Fraction 2 = 30 ml of Methanol:H_2_O (11:9, v/v): Fraction 3 = 30 ml of Methanol:H_2_O (13:7, v/v), and Fraction 4 = 50 ml of Methanol:H_2_O (7:3, v/v). A wash with 20 ml 100% methanol was used to clean the column after each elution. After the presence of the desired component was confirmed by flow injection analysis-electrospray ionization MS in negative ion mode, the solvent was evaporated and all of the material collected for Fraction 4 was pooled to obtain S4:17 for NMR analysis.

**NMR methods**

NMR data were obtained on a Varian UnityPlus spectrometer operating at 499.74 and 125.67 MHz for ^1^H and ^13^C, respectively. Sample was dissolved in 250 l of CDCl_3_ and run in a 5 mm NMR tube with Doty susceptibility matched plugs. Resonance assignments were determined by analysis of ^1^H, ^13^C, DEPT, gHMQC, gHMBC, gCOSY, and TOCSY data.
